# The Continuing and Growing Epidemic of Chronic Low Back Pain

**DOI:** 10.3390/healthcare3030838

**Published:** 2015-09-15

**Authors:** Robert J. Gatchel

**Affiliations:** The University of Texas at Arlington, Arlington, TX 76019, USA; E-Mail: gatchel@uta.edu

**Keywords:** chronic pain prevalence, low back pain, biopsychosocial model, interdisciplinary chronic pain management, illness *versus* disease

## Abstract

Because of the great prevalence of chronic pain, it is not surprising that there have been a number of influential reports by the Institute of Medicine, National Institutes of Health, and the World Health Organization that have documented the medical, social and economic problems caused by it, and the need for better pain-management programs. The present article briefly reviews these reports, and then focuses on three important areas that need to be considered when addressing the continuing and growing epidemic of one of the most prevalent types of chronic pain [chronic low back pain (CLBP)]: the biopsychosocial model of chronic pain; the paradigm shift in medicine from a disease model to an illness model of CLBP; and a review of the treatment- and cost-effectiveness of interdisciplinary chronic pain management programs. This overview will serve as an important prelude to other topics related to low back pain included in this Special Issue of *Healthcare*. Topics covered will range from assessment and treatment approaches, to important psychosocial mediators/moderators such as coping and pain beliefs.

## 1. Introduction

The very influential Institute of Medicine (IOM) Report, “*Relieving Pain in America*” [1], has highlighted the urgent need for the development of better methods for pain management because the ever-increasing costs associated with current treatment approaches cannot be sustained. This urgency has been further emphasized by the National Institutes of Health’s recent *National Pain Strategy: A Comprehensive Population Health Level Strategy for Pain* [2]. The Strategy also highlighted the use of a *biopsychosocial model of pain* (to be reviewed in the next section). This was stimulated by the initial IOM Report [1], which estimated that the total direct and indirect costs of chronic pain to the U.S. economy ranges between $ 560 to $ 630 billion annually. This amount excludes those adults in the military, VA Health Care System, incarcerated individuals, and those hospitalized in psychiatric facilities [3]. Moreover, 100 million American adults have some form of chronic pain, and it is also common among children and adolescents. Overall, this makes chronic pain more common than the total number of individuals in the U.S. with diabetes, heart disease, and cancer combined [4]! However, because most people with chronic pain do not die, it does not get the public attention it greatly deserves, and is often overlooked by federal and philanthropic funding agencies. However, as will be reviewed below, it affects a tremendous number of individuals around the world.

The IOM Report also documented that musculoskeletal pain is the most common single type of chronic pain; chronic low back pain is the most prevalent in this category. A recent article in the *Journal of the American Medical Association* reported that low back pain is one of the major health problems in the U.S., and is associated with the largest number of years lived with disability [5]. Moreover, as noted by Turk [3] in 2008, there were more than 7.3 million emergency hospital room visits, and more than 2.3 million hospital inpatient stays, that were related to back problems [6]. Globally, similar findings have been published in recent reviews in the *New England Journal of Medicine* [7] and *The Lancet* [8]. These reviews were based on the World Bank and World Health Organization’s Study of the Global Burden of Disease (GBD). As a follow-up to the previous GBD Study 2010, a more recent GBD Study 2013 [9] reported that years lived with disability (YLDs) are increasing due to population growth and aging in most countries around the world. As noted: “Leading causes of YLDs included low back pain and major depressive disorder among the top ten causes of YLDs in every country.” (p. 1) [9]. Again, the economic burden of low back pain is quite large, and continues to grow in the U.S., as well as internationally [1,9].

It should also be kept in mind that, with the “graying of America,” this low back pain problem will significantly increase in the future. In 2010, there were approximately 40.3 million Americans, age 65 years or older, accounting for 13% of the total population [10]. By the year 2030, it is projected that about 20% of the population will be 65 years of age or older [11]. Awareness of these population trends, both nationally and internationally, contributes to increased concern about healthcare issues among older adults, including pain problems, their psychiatric sequelae, and the associated increased and potentially dangerous opioid medication use.

With the above staggering statistics in mind, it was felt that a Special Issue of the Journal *Healthcare* was warranted in order to update many of the recent advances and perspectives in this growing area of clinical and economic importance. Besides the now most widely accepted and heuristic approach to chronic low back pain—*the biopsychosocial perspective*—to be reviewed next, a host of biopsychosocial-related topics will be presented. They range from medical evaluations and other assessment techniques, to low back pain management approaches, including surgery and opioid medication, as well as important psychosocial mediators/moderators such as coping and pain beliefs. An earlier review by Gatchel, Peng *et al.* [12], delineated a number of such moderators and mediators (e.g., emotional distress, catastrophizing, fear avoidance). This Special Issue is meant to provide readers with the most updated information on these important topics related to low back pain.

## 2. The Biopsychosocial Model of Chronic Pain

George Engel [13] first introduced the term “biopsychosocial” to medicine in the context of chronic physical illnesses. He initially highlighted the fact that many chronic illnesses were not solely caused by some specific underlying pathophysiology. Rather, lifestyle/psychosocial factors were important contributors to the maintenance and/or exacerbation of the illness process. This perspective started to replace the outdated *biomedical reductionism*, or “dualistic” perspective that mind and body function separately and independently, to the more comprehensive biopsychosocial approach to medicine (e.g., [4,14]). This biopsychosocial perspective began to be adopted by many clinical researchers in the area of pain, now viewing pain as the result of a dynamic interaction among biological, psychological and social factors that can perpetuate and even worsen the clinical presentation. The reader is referred to many relevant publications on this topic (e.g., [4,12,14,15,16,17,18,19,20]).

A major outgrowth of this biopsychosocial model of pain was the development of more comprehensive and effective interdisciplinary interventions for chronic pain in order to address both the physical and psychosocial factors involved (e.g., [4,16]). Indeed, as reviewed by Gatchel and Okifuji [17], traditional interventions for chronic pain had predominantly involved monotherapies, such as surgery, injections, and a wide array of pharmacotherapeutic approaches. However, as Turk and Gatchel [21] began to highlight, more comprehensive interdisciplinary approaches, based on the biopsychosocial model, were needed to address both the physical and psychosocial factors involved in chronic pain. This model has become very influential in the area of pain, especially with the resultant development of treatment- and cost-effective interdisciplinary pain management programs in this country [12,17], as well as other countries such as Canada [22], Denmark [23,24], France [25], Germany [26], and Japan [27]. Such programs (to be discussed next), based upon the biopsychosocial model, have been found to be the most heuristic approach to understanding and assessing chronic pain [12]. Indeed, the earlier reviewed influential IOM Report [1]; p. 35 states that: “Today, most researchers and clinicians who specialize in pain issues use the “biopsychosocial model” (denoting the combination of biological, psychological and social/family/cultural contexts of pain to understand and treat chronic pain [12]).” Further support for the use of interdisciplinary pain management as an evidence-based clinical guideline for the treatment of low back pain is the fact that Chou and colleagues [28] concluded that “…it is strongly recommended that clinicians consider intensive interdisciplinary rehabilitation with a cognitive/behavioral emphasis (strong recommendation, high-quality evidence)” (p. 1070).

## 3. Disease *versus* Illness

It should also be noted that, as originally summarized by Turk and Monarch [19], the biopsychosocial model focuses on both disease and illness, with illness being viewed as the complex interaction among biological, psychological and social factors. As they note:

“*The distinction between “disease” and “illness”* *is crucial to understanding chronic pain. Disease is generally defined as an “objective biological event” that involves disruption of specific body structures or organ systems caused by pathological, anatomical, or physiological changes…In contrast to this customary view of physical disease, illness is defined as a “subjective experience or self-attribution” that a disease is present; it yields physical discomfort, emotional distress, behavioral limitations, and psychosocial disruption. In other words, illness refers to how the sick person and members of his or her family and wider social network perceive, live with, and respond to symptoms and disability…The distinction between disease and illness is analogous to the distinction between “pain” and “nociception.” Nociception entails stimulation of nerves that convey information about tissue damage to the brain. Pain is subjective perception that results from the transduction, transmission, and modulation of sensory input filtered through a person’s genetic composition and prior learning history and modulated further by the person’s current physiological status, idiosyncratic appraisals, expectations, current mood state, and sociocultural environment.*”

(pp. 6–7) [19]

Because the biopsychosocial model of chronic pain views each individual as experiencing pain uniquely, it is important to evaluate the different dimensions of this interactive process [16]. Also, chronic pain should be generally viewed as an illness, which can be successfully managed (using comprehensive interdisciplinary pain management programs to be discussed next), but cannot often be completely cured by traditional surgical procedures or solely by medication. Indeed, this represents a significant paradigm shift from the older biomedical reductionist curative model of medical disorders, to a more pragmatic and effective biopsychosocial management model of medical disorders such as chronic pain.

## 4. Interdisciplinary Pain Management

Intensive interdisciplinary pain management programs, such as functional restoration (first developed by Mayer and Gatchel [29]), were established for patients who were experiencing the effects of significant physical deconditioning, chronic disability, and major psychosocial consequences. As outlined by both Gatchel and Okifuji [17] and Gatchel, McGeary *et al.* [4], the treatment team of such programs consists of a physician, nurse, psychologist or psychiatrist, physical therapist, and an occupational therapist. They interact on a daily basis in order to coordinate the following:

- The objective quantification of physical/functional deficits (at the beginning, during, and at the end of treatment) in order to tailor/individualize, monitor and guide physical and functional progress and gains. Indeed, one of the most frequent barriers to rehabilitation is physical deconditioning. Such deconditioning occurs when inactivity and disuse of the injured body part culminates in a general loss of function, which becomes progressively worse as the degree of disuse and immobilization increases [30]. The effects of this deconditioning may result in muscle atrophy, the development of stiff/hypomobile joints, loss of endurance and cardiovascular fitness, and an increase in muscle spasms [29].
- Likewise, psychosocial evaluations are conducted to aid in the tailoring of treatment for each patient, as well as to guide and monitor progress and gains.
- These above psychosocial evaluations are used in a multimodal pain and disability program, using cognitive-behavioral therapy (CBT) approaches. As previously reviewed by Gatchel and colleagues [31], CBT is a major component of interdisciplinary treatment: “The central aims of CBT are to identify and replace maladaptive patient cognitions, emotions, and behaviors with more adaptive ones in the hope of maximizing the benefits of other interdisciplinary care components (e.g., physical therapy) and increasing functional capacity through improved coping…CBT has emerged as the psychosocial treatment of choice for chronic pain.” (pp. 124–125) [31].
- Psychopharmacological interventions are also often used for detoxification purposes, as well as for psychosocial management purposes.
- Regular, ongoing interdisciplinary, medically-directed formal team staffings are held at least on a weekly basis, as well as frequent team meetings in order to ensure that patients are progressing, and that any potential barriers to improvement are immediately addressed. This regular communication and feedback among the staff is a requisite element for ensuring successful treatment outcomes.

As noted earlier, this interdisciplinary approach has been found to be both therapeutically- and cost-effective in U.S. studies, as well as studies in other countries. Successful outcomes, such as decreases in pain and opioid medication use, increases in return-to-work and activities of daily living, and decreases in subsequent healthcare visits, are obtained after intervention. This attests to the robustness of the clinical research findings and utility, as well as its fidelity [4,17]. It should also be noted that, for more acute patients, a less intensive interdisciplinary intervention program has also been found to be therapeutically- and cost-effective [31,32,33].

## 5. Summary and Conclusions

As has been reviewed, there have been a number of recent and very influential reports from the IOM, the National Institutes of Health and the World Health Organization that have highlighted the urgent need for the development of better methods for pain and disability management because the ever-increasing costs associated with treatment approaches cannot be sustained. Musculoskeletal pain is the most common single type of chronic pain, with low back pain the most prevalent in this category. Because of this increased problem of chronic pain, there has been a great increase in the number of clinical research studies evaluating aspects of the assessment, treatment and prevention of chronic pain (see [12]). The majority of this clinical research is being guided by the biopsychosocial model of pain, which views pain as a result of a dynamic interaction among biological, psychological and social factors that can perpetuate and even worsen the clinical presentation. A major outgrowth of this biopsychosocial model of pain has been the development of more comprehensive and effective interdisciplinary interventions for chronic pain in order to address both the physical and psychosocial factors involved. Such interdisciplinary approaches to pain management have been found to be more therapeutic- and cost-effective than traditional biomedical approaches on a variety of important outcome measures. Indeed, such findings have resulted in a significant paradigm shift from the outdated biomedical approaches to chronic pain, which try to “cure” the pain by surgical or medication use (often, though, unsuccessfully), to a more comprehensive pain management approach using interdisciplinary pain management programs to help patients better manage and cope with the chronic pain and any remnants of it. Moreover, the distinction between disease and illness is crucial in understanding chronic pain. In contrast to the disease perspective, which is generally defined as looking for an objective biological event involved in the disruption of specific bodily structures or chronic systems caused by some type of pathophysiology, illness is defined as a more subjective experience or self-attribution that a disease is present and will yield physical discomfort, emotional distress and psychosocial disruption.

Finally, using this biopsychosocial “illness” approach to interdisciplinary pain management programs, such as functional restoration, have been developed for patients who are experiencing the effects of significant physical deconditioning, chronic disability and major psychosocial consequences. Also, for more acute patients, less intensive interdisciplinary intervention programs have also been found to be therapeutically- and cost-effective. In these programs, a number of psychosocial moderators and mediators (e.g., emotional stress, catastrophizing, fear avoidance) need to be taken into account. Subsequent articles in this Special Issue have been provided to update information on these variables, as well as the overall topic of low back pain.

## References

[1] Institute of Medicine of the National Academy of Science (2011). Relieving Pain in America: A Blueprint for Transforming Prevention, Care, Education, and Research.

[2] National Pain Strategy: A Comprehensive Population Health Level Strategy for Pain. http://iprcc.nih.gov/National_Pain_Strategy/NPS_Main.htm.

[3] Turk D.C., Gatchel R.J., Schultz I.Z. (2014). The Biopsychosocial Approach to the Assessment and Intervention for People with Musculoskeletal Disorders. Handbook of Musculoskeletal Pain and Disability Disorders in the Workplace.

[4] Gatchel R.J., McGeary D.D., McGeary C.A., Lippe B. (2014). Interdisciplinary chronic pain management: past, present and the future. Am. Psychol. Spec. Issue Psychol. Chronic Pain.

[5] U.S. Burden of disease collaborators (2013). The State of U.S. Health, 1990–2010: Burden of diseases, injuries, and risk factors. JAMA.

[6] Agency for Health Research and Quality Healthcare Cost and Utilization Project. http://www.hcup-us.ahrq.gov/reports/statbriefs/sb105.jsp.

[7] Murray C.J.L., Lopez A.D. (2013). Measuring the global burden of disease. N. Engl. J. Med..

[8] Vos T., Flaxman A.D., Naghavi M., Lozano R., Michaud C., Ezzati M., Shibuya K., Salomon J.A., Abdalla S., Aboyans V. (2012). Years Lived with Disability (YLDs) for 1160 sequelae of 289 diseases and injuries 1990–2010: A systematic analysis for the global burden of disease study 2010. Lancet.

[9] Global Burden of Disease Study 2013 Collaborators (2015). Global, Regional, and National Incidence, Prevalence, and Years Lived with Disability for 301 Acute and Chronic Diseases and Injuries in 188 Countries, 1990–2013: A Systematic Analysis for the Global Burden of Disease Study 2013. The Lancet.

[10] U.S. Census Bureau (2011). The Older Population: 2010.

[11] U.S. Census Bureau (2000). Population Projections of the United States by Age, Sex, Race, Hispanic Origin, and Nativity: 1999 to 2000.

[12] Gatchel R.J., Peng Y., Peters M.L., Fuchs P.N., Turk D.C. (2007). The biopsychosocial approach to chronic pain: Scientific advances and future directions. Psychol. Bull..

[13] Engel G.L. (1977). The need for a new medical model: A challenge for biomedicine. Science.

[14] Gatchel R.J. (2004). Comorbidity of chronic mental and physical health disorders: The biopsychosocial perspective. Am. Psychol..

[15] Feinberg S.D., Brigham C.R., Deer T.R., Leong M.S., Buvanendran A., Gordin V., Kim P.S., Panchal S.J., Ray A.L. (2013). Assessing Disability in the Pain Patien. Comprehensive Treatment of Chronic Pain by Medical, Interventional, and Integrative Approaches: The American Academy of Pain Medicine Textbook on Patient Management.

[16] Gatchel R.J. (2005). Clinical Essentials of Pain Management.

[17] Gatchel R.J., Okifuji A. (2006). Evidence-based scientific data documenting the treatment and cost-effectiveness of comprehensive pain programs for chronic nonmalignant pain. J. Pain.

[18] Gatchel R.J., Turk D.C. (1996). Psychological Approaches to Pain Management: A Practitioner’s Handbook.

[19] Turk D.C., Monarch E.S., Gatchel R.J., Turk D.C. (2002). Biopsychosocial Approaches on Chronic Pain, in Psychological Approaches to Pain Management: A Practitioner’s Handbook.

[20] Dworkin S.F., von Korff M.R., LeResche L. (1992). Epidemiological studies of chronic pain: A dynamic-ecologic perspective. Ann. Behav. Med..

[21] Turk D.C., Gatchel R.J. (2002). Psychological Approaches to Pain Management: A Practitioner’s Handbook.

[22] Corey D.T., Koepfler L.E., Etlin D., Day H.I. (1996). A limited functional restoration program for injured workers: A randomized trial. J. Occup. Rehabil..

[23] Bendix A.E., Bendix T., Vaegter K., Lund C., Frølund L., Holm L. (1996). Multidisciplinary intensive treatment for chronic low back pain: A randomized, prospective study. Cleveland Clin. J. Med..

[24] Bendix T., Bendix A. Different training programs for chronic low back pain—A randomized, blinded one-year follow-up study. Proccedings of International Society for the Study of the Lumbar Spine.

[25] Jousset N., Fanello S., Bontoux L., Dubus V., Billabert C., Vielle B., Roquelaure Y., Penneau-Fontbonne D., Richard I. (2004). Effects of functional restoration *versus* 3 hours per week physical therapy: A randomized controlled study. Spine.

[26] Hildebrandt J., Pfingsten M., Saur P., Jansen J. (1997). Prediction of Success from a Multidisciplinary treatment program for chronic low back pain. Spine.

[27] Shirado O., Ito T., Kikumoto T., Takeda N., Minami A., Strax T.E. (2005). A novel back school using a multidisciplinary team approach featuring quantitative functional evaluation and therapeutic exercises for patients with chronic low back pain. Spine.

[28] Chou R., Shekelle P. (2010). Will this patient develop persistent disabling low back pain?. J. Am. Med. Assoc..

[29] Mayer T.G., Gatchel R.J. (1988). Functional Restoration for Spinal Disorders: The Sports Medicine Approach.

[30] Mayer T.G., Polatin P.B., Mayer T.G., Gatchel R.J., Polatin P.B. (2000). Tertiary nonoperative interdisciplinary program: The functional restoration variant of the outpatient chronic pain management program. Occupational Musculoskeletal Disorders: Function, Outcomes & Evidence.

[31] Gatchel R.J., Polatin P.B., Noe C.E., Gardea M.A., Pulliam C., Thompson J. (2003). Treatment- and cost-effectiveness of early intervention for acute low back pain patients: A one-year prospective study. J. Occup. Rehabil..

[32] Rogerson M.D., Gatchel R.J., Bierner S.M. (2010). Cost utility analysis of interdisciplinary early intervention *versus* treatment as usual for high risk acute low back pain patients. Pain Pract..

[33] Whitfill T., Haggard R., Bierner S.M., Pransky G., Hassett R.G., Gatchel R.J. (2010). Early intervention options for acute low back pain patients: A randomized clinical trial with one-year follow-up outcomes. J. Occup. Rehabil..

